# Comparison between Cardiac CTA and Echocardiography for Assessment of Ventricular Septal Rupture Diameter and Its Effect on Transcatheter Closure

**DOI:** 10.1155/2022/5011286

**Published:** 2022-10-17

**Authors:** Tongfeng Chen, Yuhao Liu, Jing Zhang, Zirui Sun, Jiangtao Cheng, Yu Han, Chuanyu Gao

**Affiliations:** People's Hospital of Zhengzhou University, Heart Center of Henan Provincial People's Hospital/Central China Fuwai Hospital/Henan Key Laboratory of Coronary Heart Disease Control/Henan Research Center for Cardiovascular Epidemiology, Zhengzhou 450003, China

## Abstract

**Objective:**

This study is aimed at comparing cardiac computed tomographic angiography (CTA) with echocardiography in the assessment of ventricular septal perforation diameter.

**Methods:**

A total of 44 ventricular septal rupture (VSR) patients undertaking transcatheter occlusion were included and randomly divided into the CTA group and echocardiography group with a 1 : 1 ratio. Clinical data, operation-related data, and 30 d follow-up data were collected and analyzed.

**Results:**

Incidence of closure failure, occluder displacement, poor occluder molding, and occluder waist diameter shrinkage between the two groups were not statistically different. The mean residual shunt volume in the echocardiography group (4.2 (3.1, 5.9) mm) was significantly higher than that in the CTA group (2.1 (0, 4.0) mm) with a *p* value of 0.005. However, no significant differences were found in all-cause mortality and incidence of operative complications within 30 days after surgery. Within the CTA group, the correlation was strongest between postoperative occluder diameter and long diameter measured by CTA with a correlation coefficient of 0.799 and *p* < 0.001, followed by the correlation between postoperative occluder diameter and mean diameter measured by CTA with a correlation coefficient of 0.740 and *p* < 0.001. The diameter measured by echocardiography was not correlated to postoperative occlude diameter.

**Conclusion:**

Assessment of VSR diameter by cardiac CTA is more accurate than by echocardiography.

## 1. Introduction

Ventricular septal rupture (VSR) is a rare mechanical complication of acute myocardial infarction (AMI) with a high mortality rate. Mortality within 1 week ranges from 67% to 82% with conservative medical treatment and the one-year survival rate is only 7% [1, 2]. Interventional closure of VSR has received increasing attention because of its minimally invasive and higher short-term survival rate, and its safety and effectiveness have been gradually verified in clinical practice [3]. However, due to loose tissue or irregular shape around the perforation and weak edges such as bulging tumors, conventional echocardiography cannot always accurately assess the effective diameter of the rupture, resulting in occluder displacement or residual shunt after interventional occlusion among VSR patients, further affecting the prognosis [4].

With the development of computed tomography (CT), scanning speed and resolution have been greatly improved, mainly reflected in the field of structural heart disease such as transcatheter aortic valve replacement [5]. In clinical settings, we have observed clearer regional anatomy in cardiac computed tomographic angiography (CTA) than in echocardiography. This study is aimed at comparing cardiac CTA with echocardiography in the assessment of ventricular septal rupture diameter and its effect on transcatheter closure.

## 2. Materials and Methods

### 2.1. Study Population

This is a prospective, single-center, randomized controlled study. A total of 67 VSR patients undertaking transcatheter occlusion in Fuwai Central China Cardiovascular Hospital were screened from June 2020 to December 2021. The inclusion criteria were as follows: (1) diagnosis of AMI according to the fourth edition of the Global Definition of Myocardial Infarction and diagnosis of VSR by echocardiography or ventriculography after AMI, (2) patients with stable vital signs after drug therapy including vasoactive drugs or cardiac assist device therapy and with tolerance for surgery until 3 weeks after MI, and (3) patients or their legal guardian have signed the informed consent. Patients were excluded if (1) they had a VSR diameter greater than 22 mm as assessed by echocardiography or cardiac CTA, or they had comorbid ventricular septal dissection and were unsuitable for interventional occlusion therapy; (2) they had uncontrolled preoperative 24-hour cardiac insufficiency and were unable to lie supine for more than half an hour; and (3) they had comorbid with other diseases requiring surgical treatments. Of all 67 VSR patients, 2 cases had three-vessel coronary heart disease and needed surgical thoracotomy, 1 case had comorbidity of giant ventricular septal dissection and was not suitable for interventional therapy, and 20 cases deteriorated and family members asked for discharge or inhospital death. A total of 44 patients were finally included for further analysis and were treated with interventional therapy 3 weeks after MI. The institutional review board of Fuwai Central China Cardiovascular Hospital approved this study ((2020) Ethical Review No. (3)), and informed consent was obtained from all patients.

Patients were divided into the echocardiography group and CTA group with a 1 : 1 ratio. Both groups received interventional occlusion 3 weeks after MI and transthoracic echocardiography within 24 hours before the operation. In addition, cardiac CTA was conducted within 72 hours before the operation in the CTA group. Echocardiography and chest X-ray were reexamined after the operation, or cardiac CTA was performed if the chest X-ray could not show the image of the occlude clearly.

### 2.2. Interventional Procedure

All procedures referred to the Chinese expert consensus on interventional therapy for common congenital heart diseases [6]. The VSR occlusion umbrella (Shanghai Shape Memory Alloy Co., Ltd.) has a left edge of 7 mm, a right edge of 3 mm, and a height of 10 mm (A7B3-10). The diameter of the occluder was 8 to 12 mm larger than the perforation diameter measured by echocardiography or CT, increased or decreased as appropriate according to the diameter size or the myocardial tissue weakness around the defect.

### 2.3. Data Collection

Patients' data included (1) demographic features such as age and gender; (2) medical history: coronary heart disease, hypertension, diabetes, cerebral infarction, hyperlipidemia, etc.; (3) heart rate, blood pressure, and laboratory test indicators at admission; (4) preoperative VSR diameter measured by echocardiography or CTA; (5) postoperative residual shunt and occluder position; and (6) postoperative occluder diameter (POD).

Methods for measuring VSR diameter by CTA: under the horizontal and coronal plane, the VSR was scanned layer by layer to find the section that could show the largest defect. Measure the diameter of the defect and obtained it in the two sections. The larger one was the long diameter by CT (CTLD), the smaller one was the short diameter by CT (CTSD), and the average value of the two was the mean diameter measured by CT (CTMD).

**Figure 1:**
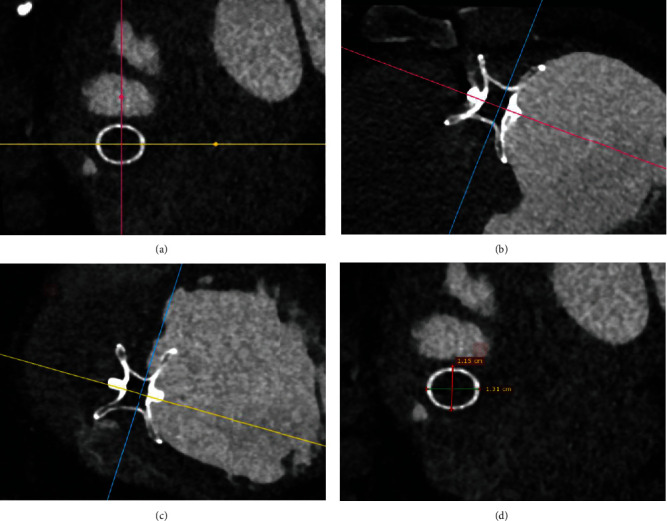
Measurement of POD by 3D-MPR mode of cardiac CT. (a) The finest part of waist in the short-axis section of occluder. (b, c) The long-axis sections of occluder perpendicular to each other. (d) The measurement method of POD, which is the average value of diameter in two vertical directions of occluder in (a).

Methods for measuring POD by CT: using 3D-MPR mode, adjust the occlude to the center of three-dimensional section so that one section tangentially cut the finest part of the short axis waist of occluder, while the other two sections vertically tangent the center of the long axis of occluder, respectively. Then, measure the diameter of the short axis waist as shown in Figure 1. The POD was the average of the diameters in two vertical directions.

### 2.4. Outcome Measures

Patients were followed up by telephone 30 days after surgery for their survival status. Primary endpoint indicators were the rates of closure failure, occluder displacement, poor occluder molding, and compression rate of the occlude waist diameter (CROWD). Secondary endpoint indicators were all-cause mortality and incidence of procedural complications within 30 days after surgery. Procedural complications included significant residual shunt (i.e., ≥5 mm diameter), valvular injury, reoperation (i.e., reclosure or surgical thoracotomy), major adverse cardiovascular events (MACE), vascular injury, hemolysis, pericardial tamponade, and high-grade atrioventricular block.

Occluder displacement was defined as the edge of either side of the occluder partially dislodged into the interventricular septum with a residual shunt > 5 mm. Poor occluder molding was defined as either side of the occluder cannot fully expand, or the CROWD is >50%. CROWD was calculated as (occluder original diameter–postoperative occluder diameter)/occluder original diameter. Valvular injury was identified if any of the following appeared: (1) new valve tendon rupture, leaflet prolapse, or obstruction of the valve by the occluder indicated by echocardiography; (2) presence of severe postoperative valve regurgitation, with mild/no valve regurgitation before the operation; and (3) presence of severe postoperative valve regurgitation, with moderate valve regurgitation before the operation, along with the regurgitation area increased by more than 5 cm^2^.

### 2.5. Statistical Analysis

Continuous variables were expressed as mean ± standard deviation or median (interquartile range) for normally distributed and nonnormally distributed ones, separately. Numerical differences between the two groups were assessed by chi-square test for categorical variables and *t*-test or Mann–Whitney *U* test for continuous variables. Scatterplots and Pearson correlation analysis were used to compare the correlation between the postoperative occluder diameter and VSR diameter measured by echocardiography or CTA. The threshold for significance was *p* = 0.05. All statistical analyses were conducted using SPSS, version 22.0 (SPSS Inc., Chicago, IL, USA).

## 3. Results

### 3.1. Baseline Comparison between the Two Groups

A total of 44 VSR patients were included in this study. There were 15 (68.2%) females in echocardiography group with an age of 68.6 ± 6.3 years and 11 (50.0%) females in the CTA group with an age of 66.7 ± 10.0 years. Compared with the CTA group (207.5 (143.0, 263.0)), the echocardiography group (282.5 (240.0, 309.0)) had significant higher platelet count with *p* value 0.023. No other significant differences were found between the two groups in the baseline, see Table 1 for details.

### 3.2. Comparison of Coronary Artery Lesions and VSR between the Two Groups

Coronary artery lesions were mainly double-vessel and triple-vessel lesions and fewer single-vessel lesions in both groups without significant difference (*p* = 0.822). Anterior descending branch was a large majority of target vascular lesions, accounting for 86.4% and 68.2% in the echocardiography and CTA groups, respectively (*p* = 0.150). The time from AMI to closure is 22 days for the echocardiography group and 23.5 days for the CTA group, but there is no statistical difference (*p* = 0.092). In addition, no significant differences were found in other variables between the two groups, see Table 2 for details.

### 3.3. Comparison of Endpoints between the Two Groups

12 (54.5%) cases and 8 (36.4%) cases in echocardiography and CTA groups had primary outcomes, respectively, resulting from the lower rate of closure failure and occluder displacement in the CTA group, but the difference was not significant (*p* = 0.226). Among them, 2 cases of closure failure in the echocardiography group were due to the fact that the occluder could not be fixed after release, and the operation was abandoned after retrieval, without occluder detachment. The CROWD between the two groups was also not statistically different (*p* = 0.546).

As for secondary endpoint indicators, 14 (63.6%) cases and 8 (36.4%) cases in the echocardiography and CTA groups had secondary outcomes, respectively, but the difference was not significant (*p* = 0.070). Although the incidence of massive residual shunt was not significantly different between the two groups (*p* = 0.082), the mean residual shunt was significantly higher in the echocardiography group (4.2 mm) than in the CTA group (2.05 mm) with a *p* value of 0.005. No statistical differences were found in 30 d all-cause mortality, valvular injury, reoperation, MACE, and vascular injury between the two groups, see Table 3 for details. No hemolysis, pericardial tamponade, or high-grade atrioventricular block occurred in either group.

We analyzed the relationship between procedural complication and 30 d mortality. A total of 17 procedural complications (11 in echocardiography group and 6 in CTA group) occurred within 30 days after the operation, with 8 cases dying. However, among 27 cases without surgical complications, only 5 cases died. The presence of procedural complications could significantly increase 30 d mortality with a *p* value of 0.043(8/17 vs. 5/27).

### 3.4. Accuracy Comparison among Different Measurements Intra-CTA Group

**Figure 2:**
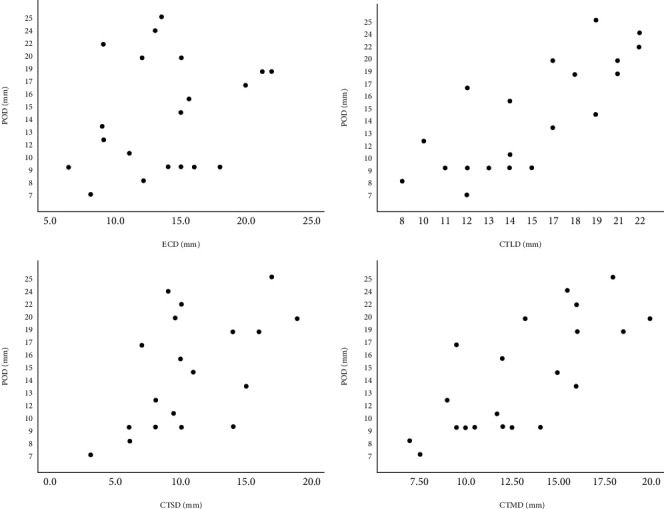
Scatter diagram of POD and ECD/CTLD/CTSD/CTMD.

As POD could best accurately reflect the true size of VSR, the accuracy criteria of VSR diameter should be based on its correlation with POD. We collected both CTA and echocardiographic evaluations of all 22 patients in the CTA group except 1 case with poor occluder molding due to distortion. The mean PDO of the remaining 21 patients was 14.9 ± 5.4 mm. Scatter diagram was developed for the relationship between POD and ECD/CTLD/CTSD/CTMD (Figure 2). The Pearson correlation test was performed between the POD and each measurement method of echocardiography and CTA, respectively, and it was found that the POD had the highest correlation with the CTLD with a correlation coefficient of 0.799 (*p* < 0.001), followed by CTMD with a correlation coefficient of 0.740 (*p* < 0.001), shown in Table 4. No correlation was found between POD and ECD.

### 3.5. Effect of Different Perforation Sites and Moderator Bands on the Postoperative Occluder Diameter

**Figure 3:**
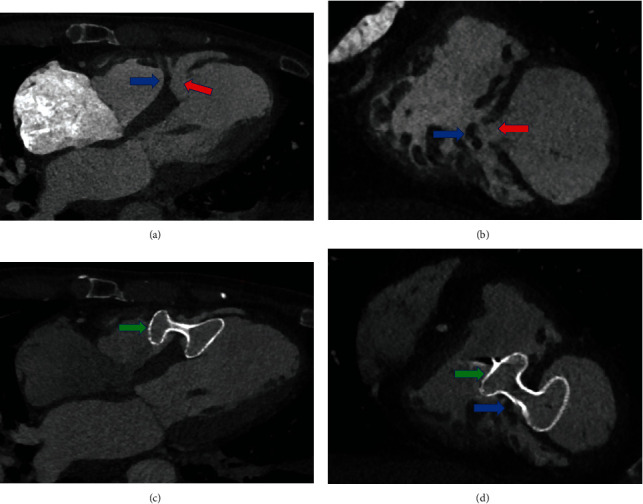
(a) Preoperative horizontal plane imaging by cardiac CTA. (b) preoperative coronal plane imaging by cardiac CTA, showing that the right ventricular surface defect has the moderator band barrier. (c) Postoperative horizontal plane imaging by cardiac CTA. (d) Postoperative coronal plane imaging by cardiac CTA, showing that the occluder waist diameter is severely compressed due to the moderator band barrier. The red arrow in the figure indicates the VSR position, the blue arrow indicates the adjustment band, and the green arrow indicates the occluder.

As shown in Figure 3, the CROWD in some anterior septal perforation patients was higher after the operation, and the POD was smaller than the defect diameter measured by echocardiography or CTA, which might be related to the thick moderator bands blocking the defect outlet in these patients. As for posterior septal rupture patients, the perforation was far away from the moderator bands and no right ventricular surface obstruction was observed. According to the perforation site suggested by CTA, 21 patients in the CTA group who could accurately measure the POD were divided into the anterior septal perforation group (*n* = 15) and posterior septal perforation group (*n* = 6). Furthermore, according to whether there was an obstruction such as an adjustable bundle at the outlet of right ventricular surface defect, they were divided into the right ventricular surface obstruction group (*n* = 7) and the no right ventricular surface obstruction group (*n* = 8).

When comparing the effect of different perforation sites and right ventricular surface obstruction on occluder molding, no significant differences were found between anterior or posterior septal perforation in POD/CTLD/POD-CTLD/occluder diameter and CROWD. However, in the intra-anterior septal group, right ventricular surface obstruction patients had a higher CROWD compared with no right ventricular surface obstruction patients (0.51 ± 0.06 vs. 0.24 ± 0.13, *p* < 0.001), although the size of the occluder was chosen by the same principle. It is because that if the right ventricular surface was obstructed, the waist of the occluder POD could not be fully stretched and resulting in the POD being much smaller even smaller than the CTLD. We can see that there is a significant difference in different values of POD and CTLD (−3.3 ± 1.5 mm vs. 1.3 ± 3.2 mm, *p* = 0.004), as Table 5 showed.

## 4. Discussion

VSR is one of the most serious mechanical complications of AMI. Although timely intravenous thrombolysis or emergency PCI can reduce the incidence of VSR, VSR still has extremely high mortality with 60-70% patients dying in 2 weeks and 3-month survival rate less than 10% [6]. Surgery serves as the main treatment for VSR, but with its invasion, high perioperative mortality rate, and 30-day mortality rate of 47% [7], the surgical cure rate of VSR remains low. In recent years, with the development of interventional technology, interventional occlusion of VSR has gradually replaced surgical thoracotomy in some experienced centers due to its advantages of being less invasive, rapid recovery, and easily tolerated, becoming the mainstream of VSR treatment. Through retrospectively analyzing 69 VSR patients who underwent interventional therapy in our center, the procedural success rate was 94.2% and a 30-day mortality rate was 23.2% [4].

In accordance with the previous experience of our center, the selection of occluder size is mostly based on the VSR size measured by echocardiography. However, in clinical practice, there always appear occluder sizes too large or too small, resulting in poor postoperative occluder molding or displacement. The reason may be related to the incomplete organization of ischemic necrosis of myocardial tissue around the rupture, or the limitations of echocardiography in the evaluation of VSR. First, the local structure is complex because of the process of tissue necrosis, absorption, and organization around the perforation, with noncircular smooth section. Second, the ventricular septum around the perforation is weak or combined with a ventricular septal aneurysm, and the size of the soft edge cannot be accurately determined by echocardiography. Third, limited by the patient's chest wall barrier and pleural gas-attenuated sound waves, the VSR section measured by echocardiography is limited and vague, and three-dimensional imaging cannot be performed. High-quality cardiac CTA can overcome the above difficulties in terms of its clear imaging and its ability to accurately assess multiple angles and planes of VSR, which can provide a more exact reference for occluder size selection.

In this study, 44 VSR patients were assessed by either echocardiography or CTA. Except for platelet count, no other significant differences were found between the two groups in baseline, coronary lesions, and perforation characteristics. Besides, no significant differences in primary outcomes were found between the two groups. As for the secondary outcomes, except for the mean residual shunt volume, no other statistical differences were found between the two groups in 30-day mortality and procedural complications. Thus, the clinical endpoint indicators of CTA had some advantages over echocardiography despite no observed significant differences, which might be related to the small sample size. We believe more significant results could be detected with a larger sample size.

The advantages of clinical outcome measures in the CTA group were mainly due to a more precise assessment of VSR diameter. In this study, the POD was used as the criterion for judging the true diameter of VSR. Since the myocardial tissue itself has certain elasticity and the traction effect of occluder, the POD may be slightly larger than the true diameter of VSR, so we considered the correlation between each measurement value and the POD as the evaluation criteria rather than the numerical proximity to ensure the reliability of. Through the scatter plot and Pearson correlation test, it was found that CTLD had the highest correlation with POD with a correlation coefficient of 0.799 (*p* < 0.001), followed by the CTMD with a correlation coefficient of 0.740 (*p* < 0.001). There was no significant correlation between ECD and POD. Therefore, CTA is more accurate in the assessment of VSR diameter, and when selecting the occluder size, CTLD should be used as the standard parameter while CTMD should be referenced as well.

The choice of occluder size should also pay attention to the VSR site and the obstruction of right ventricular surface. This study proved that although there is no difference between anterior and posterior septal ruptures in POD or CROWD, right ventricular surface obstruction patients had higher CROWD and smaller POD. Therefore, when selecting occluder size for the anterior septal perforation, it is necessary to focus on observing whether there is a thick moderator band obstructing the right ventricular surface. If so, it is feasible to select the occluder with a size of 3-4 mm smaller than the conventional choice, to avoid poor occluder molding. Moreover, we observed that the POD in patients without right ventricular surface obstruction in the anterior septal perforation was 1.3 mm larger than the CTLD, and the POD in patients with posterior septal perforation was basically consistent with CTLD, suggesting that the anterior septal perforation without right ventricular surface obstruction had better elasticity; thus, attention should be paid to such characteristics when selecting the occluder size for these two types of VSR patients.

There are some limitations to this study. First, the sample size was relatively small, which might lead to many nonsignificant differences. Second, the study had a short follow-up time and no long-term data was collected. Thus, further study on verification of the current results is required with a larger sample size and a longer follow-up time.

In conclusion, this study preliminarily verified the accuracy of cardiac CTA in evaluating VSR diameter compared with echocardiography, while the evaluation needed to be in combination with different VSR sites and right ventricular surface obstruction.

## Figures and Tables

**Table 1:** Baseline comparison between the echocardiography group and CTA group.

Items	Echocardiography group (*n* = 22)	CTA group (*n* = 22)	*p* value
Age (years)	68.6 ± 6.3	66.7 ± 10.0	0.453
Female (*n* (%))	15 (8.2)	11 (50.0)	0.220
History of coronary heart disease (*n* (%))	6 (27.3)	2 (9.1)	0.118
History of hypertension (*n* (%))	15 (68.2)	9 (40.9)	0.069
History of diabetes (*n* (%))	6 (27.3)	6 (27.3)	1.000
History of cerebral infarction (*n* (%))	1 (4.6)	1 (4.6)	1.000
History of hyperlipidemia (*n* (%))	2 (9.1)	1 (4.6)	0.550
Heart rate (beats/min)	98.5 (86.0, 110.0)	87.5 (77.0, 100.0)	0.149
Systolic blood pressure (mmHg)	111.7 ± 16.2	102.3 ± 15.0	0.052
CRP (mg/L)	45.7 (22.0, 93.2)	19.3 (3.79, 55.1)	0.120
White blood cell count (10^9^/L)	11.9 ± 5.6	11.1 ± 5.2	0.629
Hemoglobin (g/L)	120.2 ± 15.9	122.8 ± 19.0	0.627
Platelet count (10^9^/L)	282.5 (240.0, 309.0)	207.5 (143.0, 263.0)	0.023
NT-ProBNP (ng/L)	6070.5 (2056.0, 12009.0)	5288.0 (2278.0, 11309.0)	0.851
ALT (U/L)	33.8 (20.0, 142.0)	41.4 (30.0, 118.7)	0.379
AST (U/L)	42.0 (23.0, 187.0)	43.2 (24.0, 154.0)	0.888
Creatinine (*μ*mol/L)	90.5 (65.0, 114.0)	85.5 (67.0, 113.0)	0.963
LVEF (%)	53.3 ± 7.7	55.6 ± 8.1	0.338
PASP (mmHg)	61.2 ± 13.1	52.2 ± 18.5	0.175
LVDd (mm)	49.9 ± 7.0	51.4 ± 7.3	0.489
Ventricular aneurysm (*n* (%))	16 (72.7)	14 (63.6)	0.517
MCS application (*n* (%))	14 (63.6)	13 (59.1)	0.757

Note: NT-ProBNP: N-terminal pro-B-type natriuretic peptide; ALT: alanine aminotransferase; AST: aspartate aminotransferase; LVEF: left ventricular ejection fraction; PASP: pulmonary artery systolic pressure: LVDd: left ventricular end-diastolic dimension: MCS: mechanical circulatory support. This study included intra-aortic balloon pump (IABP) and extracorporeal membrane oxygenation (ECMO). 1 mmHg = 0.133 kPa.

**Table 2:** Comparison of coronary artery lesions and VSR between the echocardiography group and CTA group.

Items	Echocardiography group (*n* = 22)	CTA group (*n* = 22)	*p* value
Number of vessel lesions (*n* (%))			0.822
Single	5 (22.7)	4 (18.2)	
Double	8 (36.4)	10 (45.5)	
Triple	9 (40.9)	8 (36.4)	
Target vascular lesion (*n* (%))			0.150
Anterior descending branch	19 (86.4)	15 (68.2)	
Right coronary artery	3 (13.6)	7 (31.8)	
Treatment (*n* (%))			0.638
PCI	13 (59.1)	13 (59.1)	
PTCA	6 (27.3)	4 (18.2)	
Conservative treatment	3 (13.6)	5 (22.7)	
Timing of operation (*n* (%))			0.803
Emergency recanalization	4 (21.1)	5 (29.4)	
Elective (before perforation closure)	12 (63.2)	10 (58.8)	
Elective (after perforation closure)	3 (15.8)	2 (11.8)	
Perforation site (*n* (%))			0.232
Apical	15 (68.2)	10 (45.5)	
Anterior septum	3 (7.5)	3 (13.6)	
Posterior septum	4 (18.2)	9 (40.9)	
Time from AMI to closure (d)	22 (21, 24)	23.5 (21, 30)	0.092
ECD (mm)	13.9 ± 4.4	13.6 ± 4.3	0.777
CTLD (mm)	—	15.5 ± 4.1	—
CTSD (mm)	—	10.6 ± 4.0	—
CTMD (mm)	—	13.0 ± 3.7	—
Occluder diameter (mm)	25 (22, 28)	24 (22, 26)	0.943

Note: AMI: acute myocardial infarction; VSR: ventricular septal rupture; PCI: percutaneous coronary intervention; PTCA: percutaneous transluminal coronary angioplasty; ECD: diameter of rupture measured by echocardiography; CTLD: long diameter of rupture measured by CTA; CTSD: short diameter of rupture measured by CTA; CTMD: mean diameter of rupture measured by CTA. 36 patients underwent PCI while the remaining 8 patients received conservative treatments.

**Table 3:** Comparison of endpoint indicators between the echocardiography group and CTA group.

Items	Echocardiography group (*n* = 22)	CTA group (*n* = 22)	*p* value
Primary endpoint indicators	12 (54.5)	8 (36.4)	0.226
Closure failure (*n* (%))	2 (9.1)	0	0.148
Occluder displacement (*n* (%))	3 (13.6)	1 (4.5)	0.294
Poor occluder molding (*n* (%))	7 (31.8)	7 (31.8)	1.000
CROWD	0.40 ± 0.16	0.37 ± 0.15	0.546
Secondary endpoint indicators	14 (63.6)	8 (36.4)	0.070
30 d all-cause mortality (*n* (%))	8 (36.4)	5 (22.7)	0.322
Significant residual shunt (*n* (%))	8 (36.4)	3 (13.6)	0.082
Residual shunt (mm)	4.2 (3.1, 5.9)	2.05 (0, 4.0)	0.005
Valvular injury (*n* (%))	4 (18.2)	2 (9.1)	0.380
Reoperation (*n* (%))	2 (9.1)	0	0.148
MACE (*n* (%))	1 (4.5)	1 (4.5)	1.000
Vascular injury (*n* (%))	1 (4.5)	0	0.312
30 d all-cause mortality combined with procedural complications (*n* (%))	5 (22.7)	3 (13.6)	0.434

Note: MACE: major adverse cardiovascular events.

**Table 4:** Correlation between POD and different measurement methods in the CTA group.

Measurement index	Value	*R*	*p* value
POD (mm)	14.9 ± 5.4	—	—
ECD (mm)	13.8 ± 4.3	0.221	0.336
CTLD (mm)	15.5 ± 4.1	0.799	<0.001
CTSD (mm)	10.6 ± 4.0	0.536	0.012
CTMD (mm)	13.0 ± 3.7	0.740	<0.001

Note: POD: postoperative occluder diameter; ECD: diameter of rupture measured by echocardiography; CTLD: long diameter of rupture measured by CTA; CTSD: short diameter of rupture measured by CTA; CTMD: mean diameter of rupture measured by CTA.

**Table 5:** Effect of different perforation sites and right ventricular surface obstruction on occluder.

Measurement index	Anterior septal perforation				Posterior septal perforation (*n* = 6)	*p* value^b^
	Overall (*n* = 15)	Right ventricular surface obstruction (*n* = 7)	No right ventricular surface obstruction (*n* = 8)	*p* value^a^		
POD (mm)	15.1 ± 5.7	10.4 ± 2.1	19.3 ± 4.3	<0.001	14.2 ± 5.0	0.720
CTLD (mm)	16.0 ± 3.9	13.7 ± 2.0	18 ± 4.2	0.029	14.2 ± 4.7	0.370
POD-CTLD (mm)	−0.9 ± 3.4	−3.3 ± 1.5	1.3 ± 3.2	0.004	0 ± 3.1	0.594
Occluder diameter (mm)	24 (22, 26)	22 (20, 23)	26 (25, 27)	0.024	22 (18, 28)	0.663
CROWD	0.37 ± 0.18	0.51 ± 0.06	0.24 ± 0.13	<0.001	0.38 ± 0.12	0.902

Note: ^a^comparison between with and without right ventricular surface obstruction intra-anterior septal group; ^b^comparison between anterior and posterior septal rupture. POD: postoperative occluder diameter; CTLD: long diameter of rupture measured by CTA; POD-CTLD: the different values between POD and CTLD; CROWD: compression rate of the occlude waist diameter.

## Data Availability

The (data type) data used to support the findings of this study are available from the corresponding author upon request.
